# Vacuum-Deposited Bifacial Perovskite Solar Cells

**DOI:** 10.1021/acsenergylett.4c01536

**Published:** 2024-08-27

**Authors:** Abhyuday Paliwal, Kassio P. S. Zanoni, Cristina Roldán-Carmona, Nathan Rodkey, Henk J. Bolink

**Affiliations:** Instituto de Ciencia Molecular, Universidad de Valencia, Calle Catedrático José Beltrán 2, 46980 Paterna, Spain

## Abstract

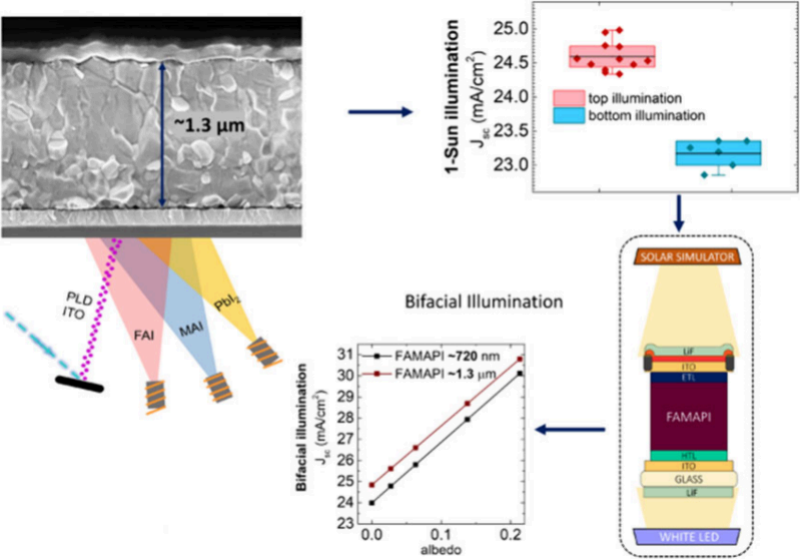

Bifacial perovskite solar cells (Bi-PSCs) require thick perovskite layers to sufficiently absorb higher wavelength light. Also, it is critical to know which electrode (top or bottom) can more efficiently harvest the direct incident solar irradiance. Here, fully vacuum-deposited Bi-PSCs are reported with perovskite layer thicknesses ranging from ∼720 nm to 1.3 μm. With an optimized ITO top-electrode, the Bi-PSCs generated higher current density under top-illumination by >1 mA/cm^2^, attaining the highest value of 24.98 mA/cm^2^. The best Bi-PSC exhibited an efficiency of 19.6% under top-illumination and 18.71% under bottom-illumination, resulting in a high bifaciality factor of ∼0.95. Furthermore, even after employing cover glass encapsulation on the top-electrode, the Bi-PSCs still produced higher current density from top-illumination. Upon bifacial illumination using simulated 1-Sun light as the main illumination and a white LED light albedo of ∼0.21, the champion Bi-PSC demonstrated a current density value of ∼30.00 mA/cm^2^.

Single junction, narrow band gap (*E*_g_ = 1.56 ± 0.02 eV) lead halide perovskite solar cells (PSCs) have rapidly advanced, attaining high power conversion efficiency (PCE > 25%) and improved device stability.^1^ The rise in the PCE of the PSCs in the recent years has been mainly a result of their increased short circuit current density (*J*_sc_ > 24 mA/cm^2^), which is primarily attained via appropriate management of the molar concentration of formamidinium lead iodide (FAPI) in the perovskite composition.^2,3^ These cells are typically fabricated in superstrate configuration which benefits from the increased absorption of the light at wavelengths close to the band edge region, as the top-metal electrode reflects the unabsorbed higher wavelength light back into the perovskite layer, thus increasing its optical path length and ensuring its increased absorption.

An alternative to the superstrate device configuration is the bifacial configuration, in which the direct sunlight is incident on one of the semitransparent electrodes and the indirect (sun) light is incident on the other semitransparent electrode.^4−6^ Depending on the type of application, bifacial solar cells can lead to a certain additional generation of photocurrent, and as a result of that, such solar cells can generate higher power density output values.^7,8^ Currently, bifacial silicon photovoltaic modules have a market share of more than 30%, and it has been predicted that this will rise to 70% by 2030.^5^ To adapt, and to target higher power-generation in perovskite devices, the community has already begun to investigate bifacial perovskite solar cells (Bi-PSCs).^4,6−10^ Recently, Jiang et. al reported Bi-PSCs having power density output of >23 mW/cm^2^ under bifacial illumination conditions.^8^ However, bifacial perovskite devices reported in the literature exhibit a low bifaciality factor of <0.95 and rely heavily on solution-based processing of the perovskite layers which leads to absorber layers that are less than 1 μm thick.^7,8^

The use of semitransparent electrodes on both sides of the device in the case of Bi-PSCs instead of only on one side as in the superstrate configuration, however, leads to absorption losses pertaining to the optical path-length, especially for the higher wavelength light.^7^ To elaborate, in an opaque superstrate PSC having one semitransparent and one metallic electrode, the optical path-length of sunlight is approximately twice as long as in a bifacial or semitransparent solar cell in which both electrodes are semitransparent. To mitigate the above absorption loss related to the optical path-length, the most obvious thing to do is to increase the thickness of the perovskite absorber layer. One of the benefits of the vapor-phase deposition of perovskites compared to its solution processing is the ease by which the film thickness can be increased via extending the deposition time or rate of deposition.^11−13^ Whereas in the solution-based processes, generally the concentration of the precursor solution needs to be adjusted to modulate the perovskite film thickness,^14^ which affects the resultant coating and crystallization processes.^15^

A second important factor for Bi-PSCs is the minimization of parasitic absorption losses from the selective charge extraction layers. This is important on both sides of the perovskite absorber layer as sunlight, or at least a part of it, enters the Bi-PSC from both sides of the device. The thickness of the charge extraction layer deposited on top of the perovskite layer depends on the roughness of the perovskite layer;^16^ it increases with the roughness of the perovskite film to ensure that there is no direct contact between the perovskite and the top semitransparent electrode.^7,8^ Vapor-phase deposited perovskite layers have a very smooth and flat surface even when the film is ∼1 μm thick, with root-mean-square (RMS) roughness values typically below 15 nm.^11^ This enables the use of thin charge extraction layer on top of the perovskite which is conducive for having lower parasitic absorption losses.^11,17^ This, together with an optically optimum or less-reflecting top-electrode, is expected to increase the *J*_sc_ generated by the Bi-PSCs when illuminated from the top-electrode, thereby pushing their bifaciality factor closer to the desired value of 1. Thermal coevaporation of the perovskite precursors is one of the most used vapor deposition processes for perovskite film deposition.^18^ Thermal evaporation method, in general, is a dry, mature, and scalable thin-film deposition technique that is widely used in the industrial production of organic semiconductor-based devices. It has also successfully demonstrated its efficacy in fabricating perovskite layers having both simple and complex compositions,^19,20^ that are compact and thin (<100 nm),^21−23^ and thick (≥1 μm)^11,13,17^ for relevant applications, and overlarge area (>50 cm^2^).^24,25^ Furthermore, in line with the current standards of industrial manufacturing, fully vacuum-deposited PSCs have been realized that exhibited respectable PCE values and moderate to high thermal stability upon stressing at high temperature of 85 °C.^19,26,27^

Here, we fabricated fully vacuum-deposited, p–i–n, Bi-PSCs with three different formamidinium methylammonium lead iodide (FAMAPI) perovskite layer thicknesses of ∼720 nm, ∼820 nm, and ∼1.3 μm. The optimal thickness of lithium fluoride (antireflection layer) and indium tin oxide (transparent conducting oxide) layers of the top-electrode (at the n-side) of the Bi-PSC were determined using optical simulations and implemented in all the devices. The *J*–*V* measurements revealed that the Bi-PSCs produced higher short circuit current density (*J*_sc_) values (>1 mA/cm^2^) when illuminated from the top-electrode (top-illumination) than bottom-electrode (bottom-illumination) with the simulated 1-Sun light. The Bi-PSC having a FAMAPI thickness of ∼1.3 μm demonstrated the highest *J*_sc_ value of 24.98 mA/cm^2^, which is the highest *J*_sc_ value ever demonstrated under top-illumination. The best Bi-PSC exhibited a PCE of 19.6% under top-illumination conditions and 18.71% under bottom-illumination, resulting in a bifaciality factor of approximately ∼0.95. Interestingly, even after employing a cover glass encapsulation at the top-electrode side of the device, the Bi-PSC still produced higher *J*_sc_ from top-illumination than bottom-illumination. Upon bifacial-illumination conditions using a white LED as the albedo (rear) illumination source, the champion Bi-PSC demonstrated *J*_sc_ (output power density in mW/cm^2^) values of 25.78 (19.60), 27.92 (21.13), and 30.07 (22.63) mA/cm^2^ under albedo of ∼0.6, ∼0.14, and ∼0.21, respectively. Both the superstrate and Bi-PSCs retained more than 80% of their initial PCE for more than 600 h when stressed at 85 °C on a hot plate inside a glovebox. This work demonstrates the efficacy of the thermal evaporation method in producing micrometer thick perovskite films for Bi-PSC application and of the top-electrode in producing remarkably high *J*_sc_ values in the Bi-PSCs under the primary AM 1.5 G monofacial illumination.

The fabrication of bifacial PSCs (Bi-PSCs) is not significantly different nor more expensive than that of the conventional superstrate PSCs, as the key differences in the two device configurations stem from the use of different top-electrode materials and their associated deposition methods.^8^ Transparent conductive oxide (TCO) materials are almost universally employed as the bottom-electrode on glass substrates in superstrate-type solar cells.^28−30^ This is because the higher thermal processing window of glass allows to obtain TCOs with high transparency and conductivity.^31,32^ Similarly, for their optical transparency and electrical conduction, TCOs are also often the choice for the top-electrode.^33^ However, in this case more care must be taken as the semiconductor layers in the solar cells have a much lower thermal window than glass, and they can easily get damaged from the high impact of the TCO deposition process thereby affecting the device performance.^33^ Here, we employed pulsed laser deposition method for depositing the top-ITO electrode during the fabrication of Bi-PSCs as it allows for a relatively soft deposition of ITO on PSC-stacks as demonstrated previously.^27^ The schematic of the device stack employed for the fabrication of Bi-PSCs is shown in Figure 1a. Formamidinium methylammonium lead iodide (FAMAPI) perovskite composition grown by a three source cosublimation process was incorporated in the p-i-n PSCs due to its relatively high thermal stability, and absorbance range.^27,34,35^ As HTL, we employed a bilayer consisting of a strong oxidant, CS-9 and the hole transporting molecule *N*4,*N*4,*N*4″,*N*4″-tetra([1,1′-biphenyl]-4-yl)-[1,1′:4′,1″-terphenyl]-4,4″-diamine (TaTm). Subsequently, the FAMAPI perovskite layer was grown by coevaporating PbI_2_, MAI, and FAI (see experimental section for more details). Importantly, the rate of MAI’s evaporation was controlled using a quartz crystal microbalance sensor that was also exposed to the evaporation flux of PbI_2_ and FAI.^36^ After the perovskite deposition, the samples were annealed at 100 °C for 10 min, and then the electron transporting layers (ETL), consisting of 15 nm fullerene C_60_ followed by 7.5 nm bathocuproine (BCP) were deposited. Next, after the top-ITO deposition by the PLD process, silver gridlines were deposited outside the active area on top of the ITO to facilitate charge extraction without incurring resistive losses. A ∼25 nm Al_2_O_3_ (alumina) was used as an encapsulation layer and deposited after the Ag gridlines using the atomic layer deposition (ALD) process as this ensures the formation of a pinhole-free layer. Finally, a LiF antireflective coating was deposited on top of the alumina layer, as well as on the other side of the glass substrate by thermal sublimation in a high vacuum chamber. Note that the ∼25 nm alumina layer above the top-ITO layer also acts as a secondary antireflection layer,^17,37^ in addition to its primary role of serving as a device encapsulation layer.

**Figure 1:**
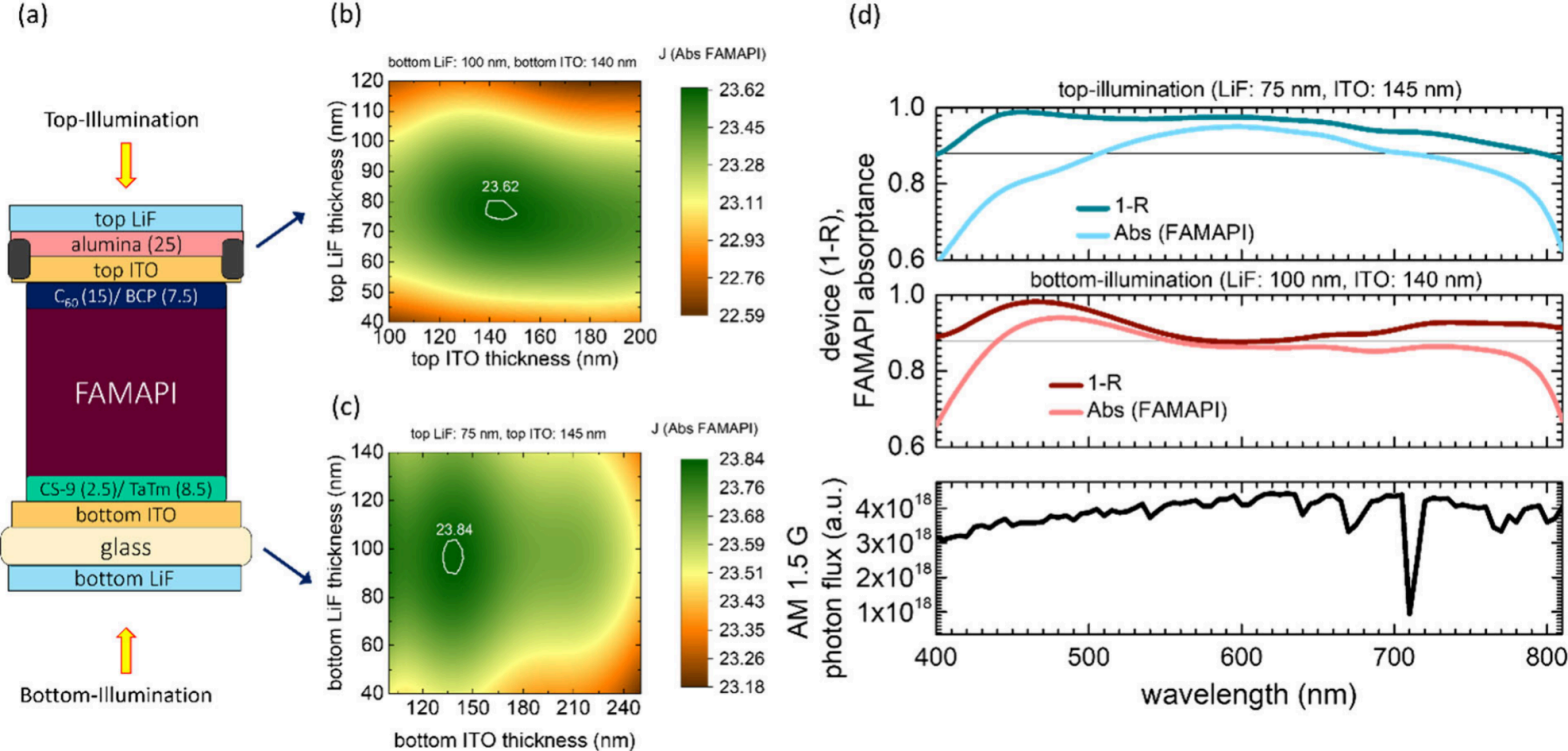
(a) Schematic of the device stack of the fully vacuum-deposited Bi-PSC fabricated in this work. (b, c) Contour plots of simulated J (Abs FAMAPI) values of the Bi-PSC when illuminated from top and bottom electrodes, respectively, as a function of LiF and ITO thicknesses. The solid lines in the two contour plots demarcate the region corresponding to their respective highest *J* (Abs FAMAPI) values. Note that optimal thickness values of bottom ITO and bottom LiF layers were used for simulating the J (Abs FAMAPI) from top-illumination, and optimal thickness values of top ITO and top LiF layers were used for simulating the *J* (Abs FAMAPI) from bottom-illumination. (d) Plots of simulated “1-R” spectrum of Bi-PSC and simulated FAMAPI’s absorptance spectrum when the Bi-PSC having optimal thicknesses of LiF and ITO at both the top and bottom electrodes is illuminated from the top-electrode (top-illumination) or bottom-electrode (bottom-illumination) as well as the AM 1.5 G photo flux spectrum, for the comparison of the simulated reflectance losses in the Bi-PSC under top and bottom-illumination conditions. The solid horizontal lines in the top and middle panels are at the same “*y*” value to aid the eye in comparing the spectra.

To capitalize on the bifacial absorption in Bi-PSCs for maximizing its *J*_sc_ and, therefore, PCE, it is imperative to (1) determine the optimum thicknesses of the LiF and ITO layers of both the top and the bottom transparent electrodes and (2) which of the two electrodes (the top or the bottom one) leads to the most efficient harvesting of the sunlight. To evaluate the former, we performed transfer matrix based optical simulations to obtain the simulated absorptance in the FAMAPI layer of the Bi-PSC under the top and bottom-illumination conditions for a range of relevant thicknesses of ITO and LiF layers at both the top and bottom-electrodes. The optical simulations were done considering a minimum ITO threshold thickness of 100 nm as the inadequate sheet resistance of a thinner (<100 nm) ITO layer is likely to incur series resistance losses in the device, especially under bifacial-illumination conditions and in large area cells.^7^ The optical constants used in the simulations of all of the layers of the stack are shown in Figure S1. The simulated absorptance spectra of the FAMAPI layer obtained from both the top and bottom-illumination of the Bi-PSC were integrated with the AM 1.5 G irradiance and mathematically treated to obtain the corresponding equivalent current density values: J (Abs FAMAPI). The contour plots of the simulated J (Abs FAMPI) values of the Bi-PSC under both top- and bottom-illumination conditions are shown in Figure 1b, c. The optimum thickness values of LiF and ITO layers for both the top and bottom-electrodes should correspond to the region (marked with solid lines) in their respective contour plots where the value of *J* (Abs FAMAPI) is the highest. Based on this, the optimum layer thicknesses of the top-electrode are LiF = 75 nm, ITO = 145 nm, and of the bottom-electrode are LiF = 100 nm, ITO = 140 nm. Note that the above optimum thickness values of the ITO and LiF layers of both the top and bottom electrodes are independent of the perovskite layer thickness in the Bi-PSC (Figure S2). For completeness, we also simulated the *J* (Abs FAMAPI) of the Bi-PSC for lower ITO thicknesses for both the top and bottom-illumination conditions to determine the “absolute” optimum thickness values of ITO and LiF layers of both the top and bottom-electrodes, and the results are shown and discussed in Figure S3.

For comparison of the reflectance losses in the Bi-PSC from the top and bottom-illumination conditions, both the simulated absorptance spectrum of the FAMAPI layer and the 1-reflectance (1-R) spectrum of the Bi-PSC corresponding to the optimum thicknesses of the ITO and LiF layers at both the top and bottom-electrodes, as well as the spectrum of the AM 1.5 G photon flux are shown in Figure 1d. The 1-R spectrum corresponds to the fraction of incident light that is not reflected (or that is transmitted into the PSC) at the initial air/PSC interface. The comparison of 1-R curves reveals that the top-illumination condition leads to relatively lower reflectance losses over a large spectrum range of ∼480–780 nm, which is also where the density of AM 1.5 G photon flux is relatively high. The above lower reflectance results in the higher absorptance of light in the FAMAPI layer in a part of the above spectrum under the top-illumination condition. Note that the highest values of the simulated *J* (Abs FAMAPI) from the top and bottom-illumination of the Bi-PSC are comparable in magnitude: 23.62 mA/cm^2^ and 23.84 mA/cm^2^, respectively (Figure 1b, c), whereas the highest simulated J (1-R) value for top-illumination is higher by ∼1 mA/cm^2^ than that of the bottom-illumination condition. Overall, considering the possibility of slight inaccuracy in the values of the optical constants used, the above results suggest that the Bi-PSC should produce higher *J*_sc_ under the top-illumination due to having lower reflectance losses relative to the bottom-illumination condition. To visualize the influence of the shape of the AM 1.5 G photon flux spectrum on the current generation, the comparison of the simulated absorptance spectrum of the perovskite as well as the 1-R profile of the Bi-PSC resulting from having optimum and slightly suboptimum ITO and LiF layer thicknesses at the two electrodes are presented in Figure S4. The very flat nature of the sublimed layers and the planar ITO substrates leads to relatively pronounced interference induced features in the “1-R” spectra such as “local absorption maxima and minima”. These interference effects would disappear if either the perovskite layer or the TCO was sufficiently rough due to scattering of the light from the rough surface(s).^38^ Vacuum-deposited perovskite layers on ITO bottom-electrodes are generally very flat and therefore, this analysis is important for the corresponding devices. The minimum sheet resistance obtained for the room-temperature processed PLD ITO film is 30 Ω/sq, which is slightly higher than the typical value of the commercially available glass/ITO substrates ( ∼10 Ω/sq), where the ITO is usually deposited by sputtering at high temperatures of >300 °C. Therefore, to overcome possible limitations in sheet resistance that would impose a drop in the fill factor of the fabricated devices, we opted on using commercially obtained patterned-ITO substrates as the bottom contacts. The employed substrates had ITO layers of fixed different thicknesses, i.e., ∼150 nm and ∼210 nm but similar sheet resistance of ∼7 Ω/□, and were obtained from different providers (hence different optical constants as well), on which we deposited a ∼100 nm LiF layer on the glass side. On the other hand, the optimum top-electrode having the optimal thicknesses of the PLD ITO and LiF layers was implemented in the fabricated Bi-PSCs.

We fabricated fully vacuum-deposited Bi-PSCs having three different thicknesses of FAMAPI absorber layers: ∼720, ∼880, and ∼1.3 μm for comparing their J_sc_ under top- and bottom-illumination conditions. We also simultaneously fabricated opaque superstrate PSCs having the same three perovskite layer thicknesses and a ∼100 nm thick LiF antireflection layer on the glass/air interface which serve as reference or control devices. The cross-sectional SEM images of the above Bi-PSCs as well as the absorbance spectra of their corresponding FAMAPI layers are shown in Figure 2a–d. The SEM images reveal a high level of planarity in the differently thick, coevaporated perovskite layers as well as conformally deposited subsequent layers in the devices. Furthermore, we can also observe both large grains extending to almost the entire perovskite layer thickness particularly in the case of the ∼720 nm thick FAMAPI layer (Figure 2a, Figure S5), as well as relatively smaller grains that are present in all three FAMAPI layers. Other material properties such as the bandgap derived from the Tauc plot and the X-ray diffraction (XRD) pattern of the FAMAPI layers are shown in Figure S6. The current density–voltage (*J*–*V*) curves of the champion Bi-PSCs under top and bottom illumination conditions and of the superstrate reference cells under simulated 1-Sun illumination are shown in Figure 2e–g. The corresponding statistics of the PCE parameters are listed in Figure S7.

**Figure 2:**
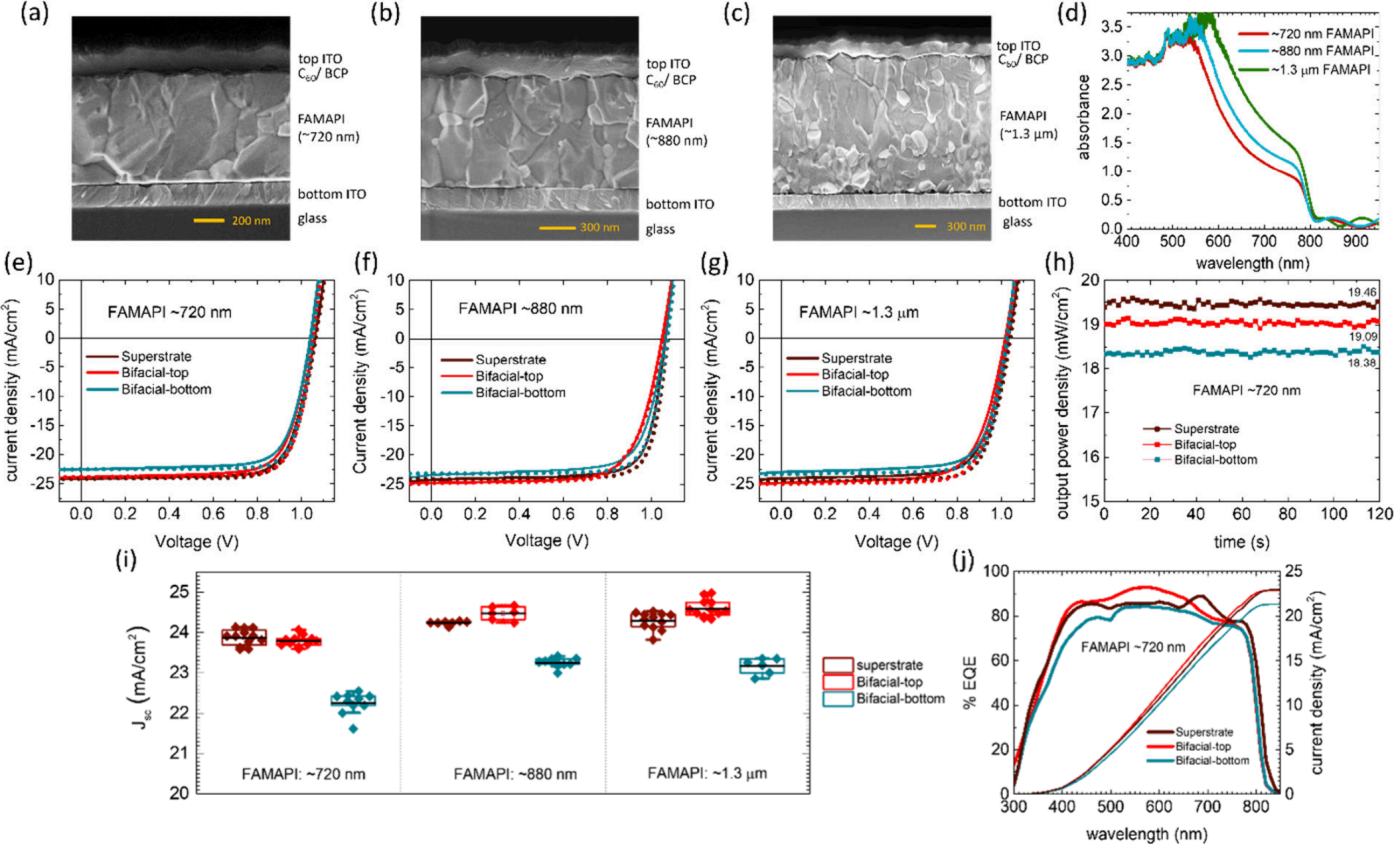
(a–c) Cross-sectional SEM images of the Bi-PSCs having FAMAPI thicknesses of ∼720 nm, ∼880 nm, and 1.3 μm, respectively. (d) Absorbance spectra of the above employed FAMAPI layers. (e–g) *J*–*V* curves under simulated 1-Sun illumination obtained from forward scan (solid) and reverse scan (dotted) of the champion Bi-PSCs and superstrate PSCs having different thicknesses of the FAMAPI layer. (h) Maximum power point tracking of the representative Bi-PSCs having ∼720 m FAMAPI layer under top and bottom-illumination conditions and the corresponding superstrate PSC. The numbers in the panel indicate the power density generated by the above PSCs at the end of 120 s. (i) Statistics of the *J*_sc_ values of the Bi-PSCs having three different thicknesses of the FAMAPI layer under top and bottom illumination conditions and the corresponding superstrate PSCs. The solid black lines in the box plots represent the mean values of the respective subsets. (j) EQE curves of the representative Bi-PSC having ∼720 m FAMAPI layer under top and bottom-illumination conditions and the corresponding superstrate PSC.

The average *J*_sc_ of the superstrate (opaque) devices, which benefit from absorption of the unabsorbed higher wavelength light that gets reflected from the top-metal electrode, is similar to that of the Bi-PSCs under top illumination when the employed FAMAPI thickness is ∼720 nm, whereas interestingly, it is higher in the Bi-PSCs having thicker FAMAPI layers under top-illumination than their respective superstrate counterparts. The highest *J*_sc_ value achieved under top-illumination condition is 24.98 mA/cm^2^ in the case of ∼1.3 μm FAMAPI-based Bi-PSCs (Figure 2i, Table S1), which is the highest value reported for a single-junction Bi-PSC from top-illumination, as well as the highest values reported for a single-junction Bi-PSC irrespective of the side of the illumination (Figure S8). Unfortunately, despite the increase in the average *J*_sc_ value of the Bi-PSCs with the perovskite thickness under the top-illumination, the Bi-PSCs having ∼880 nm, and ∼1.3 m FAMAPI layers exhibit lower FF values under the top-illumination, and as a result of this, the best performing Bi-PSC (the representative case) is obtained with a FAMAPI thickness of ∼720 nm (Figure S7, Table S1). The champion cells with this perovskite thickness give a PCE of 19.6% (stabilized power density output of 19.09%) and 18.7% (stabilized power density output of 18.38%) in the reverse-scan (open circuit voltage: *V*_oc_ to *J*_sc_) from the top and bottom-illumination conditions, respectively (Figure 2h, Table S1), which translates into a bifaciality factor (stabilized power density output ratio when illuminated from the top-electrode and bottom-electrode) of approximately ∼0.95. The higher average PCE value of the representative Bi-PSCs under top-illumination conditions than bottom-illumination is primarily due to its higher average *J*_sc_ value (by ∼1.5 mA/cm^2^) (Figure 2i) in the former case than the latter. On the other hand, the difference in the average *J*_sc_ values of the Bi-PSCs having ∼880 nm and ∼1.3 μm FAMAPI layers under top and bottom illumination conditions is of ∼1.1 mA/cm^2^ and ∼1.4 mA/cm^2^, respectively. The external quantum efficiency (EQE) spectra of the representative Bi-PSC reveal that it absorbs more light under the top-illumination condition almost across all the wavelengths due to having considerably lower reflectance losses than from bottom-illumination (Figure S9), as also indicated by the optical simulations (Figure 1d). Next, to reflect on the light harvesting efficacy of the representative Bi-PSC under top-illumination condition, we compare its EQE spectra with that of its superstrate counterpart. The comparison of their EQE spectra reveals that the Bi-PSC absorb more light than the superstrate PSCs over a large spectrum of ∼400–650 nm again due to having lower reflectance losses (Figure S9), whereas the latter absorbs more than the former only in a part of the “cavity-region” (∼650–820 nm) of absorption due to the interference effect resulting from the presence of the reflective top-metal electrode. Similar differences in the shape of the EQE spectra are also observed in the case of ∼880 nm and ∼1.3 μm FAMAPI-based bifacial and superstrate PSCs (Figure S10). The presence of the metal-top electrode in the superstrate devices also causes a small increase in the bandwidth of the absorption of the PSC as reflected by their slightly red-shifted EQE-tail relative to that of the Bi-PSCs from both top and bottom illumination conditions (Figure 2j, Figure S10). The higher EQE values of the superstrate PSCs in the cavity region, in conjunction with the higher photon flux of the AM 1.5 G irradiance, decrease the difference between the integrated *J*_sc_ values of the bifacial and superstrate devices (Figure 2d, Figure S10). Overall, the above competing effects: lower reflectance losses in Bi-PSCs under top-illumination condition vs interference induced gain in absorption at higher wavelengths in superstrate PSCs, result in comparable *J*_sc_ values when the FAMAPI thickness in the PSCs is ∼720 nm, and higher *J*_sc_ in the Bi-PSCs under top-illumination than their corresponding superstrate devices in the case of ∼880 nm and ∼1.3 μm thick FAMAPI layer-based PSCs (Figure 2i), as mentioned previously. This reflects the remarkable efficacy of the optimized top-electrode in harvesting the AM 1.5 G irradiance into the Bi-PSC.

We continued to measure the *J*–*V* characteristics of Bi-PSC under bifacial illumination conditions. A white-light LED mini panel was used as the secondary light source to illuminate the Bi-PSC through the bottom-electrode as illustrated in Figure 3a. The spectrum of the LED mini panel as well as the photographs of the bifacial-illumination apparatus are presented in Figure S11. Its intensity was calibrated using a precalibrated silicon reference diode (see method section). The *J*–*V* curves and the stabilized power density output values of the Bi-PSCs having FAMAPI thicknesses of ∼720 nm (representative) and ∼1.3 μm under bifacial illumination conditions: 1-Sun illumination through the top electrode, white light illumination with varied light intensities through the bottom-electrode, are shown in Figure 3b, c and Figure S13, respectively. Both the *J*_sc_ value and the stabilized power density output value of the Bi-PSCs increase almost linearly with the intensity of the bottom white light (Figure 3c, d and Figure S12). The *J*_sc_ and the stabilized output power density of the representative Bi-PSC having ∼720 nm FAMAPI increase from 24.00 mA/cm^2^ to 30.07 mA/cm^2^ and 18.09 mW/cm^2^ to 21.86 mW/cm^2^, respectively (Table S2), when the albedo increases from 0 to ∼0.21. Whereas in the case of thick Bi-PSC having ∼1.3 μm FAMAPI layer, the *J*_sc_ and the stabilized output power density increase from 24.83 mA/cm^2^ to 30.73 mA/cm^2^ and 18.37 mW/cm^2^ to 22.63 mW/cm^2^, respectively (Table S3), under the same conditions (Figure 3b, Figure S12).

**Figure 3:**
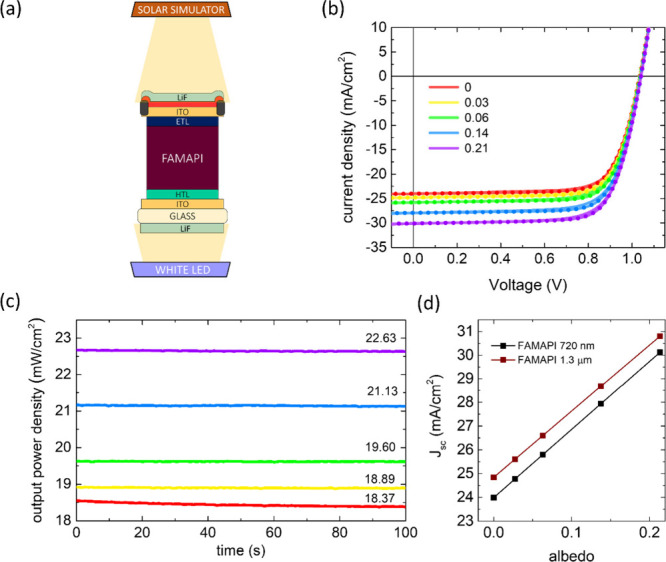
(a) Schematic of the bifacial illumination and measurement setup. The top-electrode of the bare Bi-PSC faced the primary simulated 1-sun illumination, whereas its bottom-electrode faced the white-light of the LED mini panel. (b) *J*–*V* curves obtained from forward scan (solid) and reverse scan (dotted) of the representative Bi-PSC under bifacial-illumination conditions comprising: a constant simulated 1-Sun illumination and albedo from the white-light illumination ranging from 0 to 0.21 (rounded off to second place after decimal). (c) Maximum power point tracking of the representative Bi-PSC under bifacial-illumination conditions, as described in (b). The numbers in the panel indicate the power density generated by the Bi-PSC under different bifacial-illumination conditions at the end of 100 s. (d) The *J*_sc_ values (average of forward and reverse scan) of the representative Bi-PSC as well as Bi-PSC having ∼1.3 μm FAMAPI layer under the above bifacial-illumination conditions.

The main benefit of illuminating the above Bi-PSCs from the top-electrode is that they have a higher *J*_sc_ due to lower reflectance losses originating from the use of dual antireflection layers: alumina and LiF on top of the top-ITO. For outdoor deployment, however, the above ALD-deposited alumina layer alone would be insufficient to protect the PSC against environmental and mechanical stresses, and therefore, some form of additional encapsulation must be employed. Currently, most PV technologies use glass-based or plastic-based encapsulation for protection.^39−41^ Employing any of the above encapsulation methods to the Bi-PSC, however, might compromise the transmission of light into the device stack under top-illumination as it involves incorporation of additional layers on top of the top-electrode. To determine if the top-illumination can still produce higher *J*_sc_ in our fully vacuum-deposited Bi-PSCs than bottom illumination after the implementation of a robust device encapsulation, we encapsulated a Bi-PSC sample with a cover glass using a commercial UV-curable epoxy encapsulant (Everlight’s Eversolar AB-302). Note that the above encapsulation was employed on the Bi-PSC device stack having alumina as the final layer and the LiF antireflection layer was deposited on top of the cover glass as shown in Figure 4a.^42^ The cover glass used for encapsulation has the same transmittance spectrum as a common glass substrate (Figure S13). For fairness, we used the Bi-PSC sample (one with an ∼880 nm FAMAPI layer) that previously demonstrated the lowest mean difference in the *J*_sc_ under the top- and bottom-illumination conditions. The *J*–*V* curves of champion cells, EQE spectra, as well as the statistics of the *J*_sc_ values under monofacial simulated 1-Sun illumination of the optimized bare Bi-PSC (without cover glass encapsulation) from top and bottom-illumination conditions and the cover glass encapsulated Bi-PSC from top-illumination are shown in Figure 4b–d. While the cover glass encapsulated Bi-PSC has a lower average *J*_sc_ than the bare device under the top-illumination condition, it is still higher than that of the Bi-PSC under the bottom-illumination condition (Figure 4d) due to having lower reflectance losses (Figure S14). Interestingly, the incorporation of the cover glass encapsulation does not reduce the EQE of the Bi-PSC from top-illumination across the entire spectrum of absorption but only in the small spectrum of ∼500–700 nm. Moreover, the incorporation of the above cover glass encapsulation: epoxy encapsulant/cover glass/LiF has an “antireflection” effect on the underlying device stack under top-illumination condition; the cover-glass encapsulated Bi-PSC exhibits higher *J*_sc_ and EQE than a bare Bi-PSC having alumina as the final layer under top-illumination conditions (Figure S15). This originates from the lower refractive indices of the above encapsulation layers than alumina. However, the above antireflection effect of the encapsulation layers on the underlying device stack is obviously lower than that of the optimally thick LiF layer (Figure S15).

**Figure 4:**
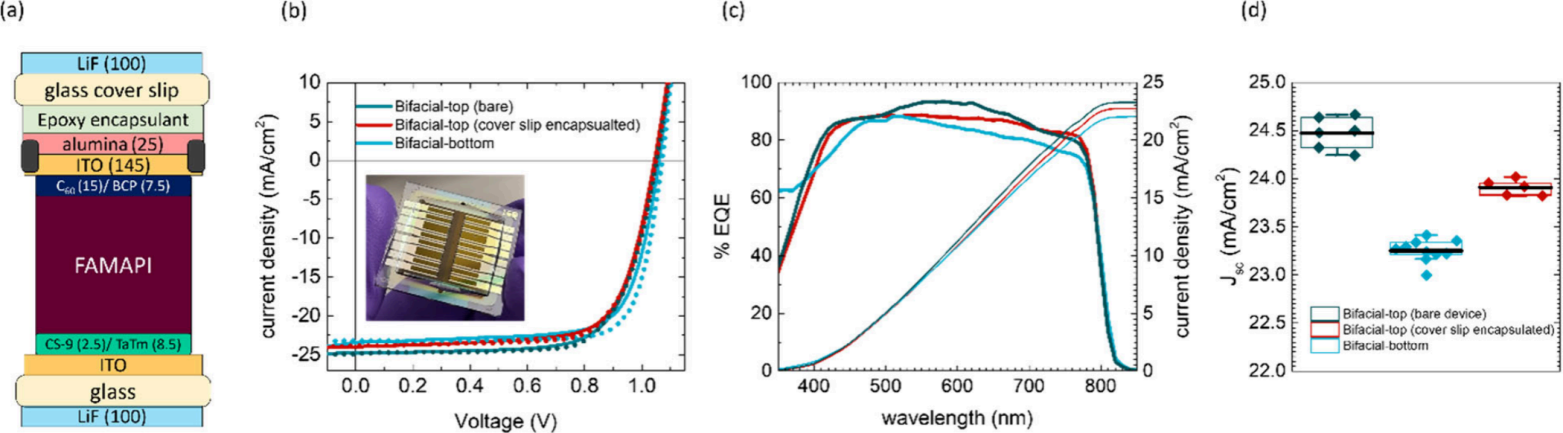
(a) Schematic of the Bi-PSC device stack after employing cover glass encapsulation. (b) *J*–*V* curves obtained in forward (solid) and reverse (dotted) scans of the champion cell of the bare Bi-PSC having ∼880 nm FAMAPI under top and bottom-illumination as well as after cover glass encapsulation under top-illumination. (c, d) Corresponding EQE spectra and statistics of the *J*_sc_ values, respectively, of the devices. Panel (c) has the same legend as panel (b). The solid black lines in the box plot of panel (d) indicate the mean values of the respective subsets. Note, the EQE of the Bi-PSC under bottom illumination is slightly different than what is shown in Figure 2J. This is caused by the use of a different batch of commercial ITO coated substrates that had a thicker ITO layer thickness (210 nm vs 147 nm).

Finally, we thermally stressed both the superstrate PSCs and Bi-PSCs on a hot plate at 85 °C in a N_2_ atmosphere to determine the thermal stability of the fully vacuum-deposited devices. Both types of devices maintained more than 80% of their respective initial efficiencies for over 600 h (Figure 5a). Interestingly, the *V*_oc_ of the two kinds of devices slightly increased up to 600 h of stressing,^19^ and the *V*_oc_ of the Bi-PSC from top-illumination which was initially comparable to that of the superstrate device slightly became higher than that of the latter in the above course of time (Figure S16). The *J*_sc_ of the superstrate devices overall decreased whereas that of the Bi-PSC increased with thermal stressing up to 600 h (Figure 5b). On the other hand, the FF of the two types of devices mainly decreased during stressing following a similar pattern, which has been previously observed as well for fully vacuum-deposited PSCs (Figure 5c).^27^ Note that the PCE evolution pattern of the two types of PSCs was very similar to that of their FF, indicating the latter to be the primary PCE-parameter that limits the device stability. To gain some insight into the underlying factor(s) responsible for the above observed evolution of the PCE-parameters upon thermal stressing, we compared the X-ray diffraction patterns of the stressed superstrate and bifacial devices with that of the pristine FAMAPI layer (Figure 5d). Preliminary observation mainly reveals that the intensity of the perovskite diffraction peaks remained almost unchanged; the intensity of the peaks only slightly increased in the case of FAMAPI layer of the superstrate device. On the other hand, a very similarly increased lead iodide’s (PbI_2_) diffraction peak at 2Θ ∼ 12.6°, and a newly appeared PbI_2_’s diffraction peak at 2Θ ∼ 38.6° are observed in the diffraction patterns of both the stressed devices.^27^ This suggests a similar increase in the quantity of the crystalline PbI_2_ phase in the FAMAPI films of both devices upon thermal stressing. To learn more about the spatial distribution of the observed crystalline PbI_2_ in the degraded PSC sample, we performed grazing incidence wide-angle X-ray diffraction (GIXRD) measurements of a pristine and a degraded FAMAPI device. The GIXRD measurements of the pristine FAMAPI device indicate that the crystalline PbI_2_ phase corresponding to the PbI_2_ peak observed in the powder diffraction measurement (Figure 5d), exists in the lower part of the coevaporated FAMAPI layer likely close to the hole transport layer (Figure 5e).^43^ On the other hand, the GIXRD measurements of the thermally stressed superstrate device reveal that the ratio of the intensity of the PbI_2_ (001) and perovskite (100) peaks only becomes greater than 1 at higher omega values. This indicates that the increased crystalline PbI_2_ phase present in the thermally stressed sample is also located at the lower part of the perovskite layer or near the buried interface (Figure 5f). From this we infer that most likely the buried interface is the stability limiting one in the p–i–n PSCs, when the coevaporated perovskite layer is grown on top of aryl amine-based hole transport layers.

**Figure 5:**
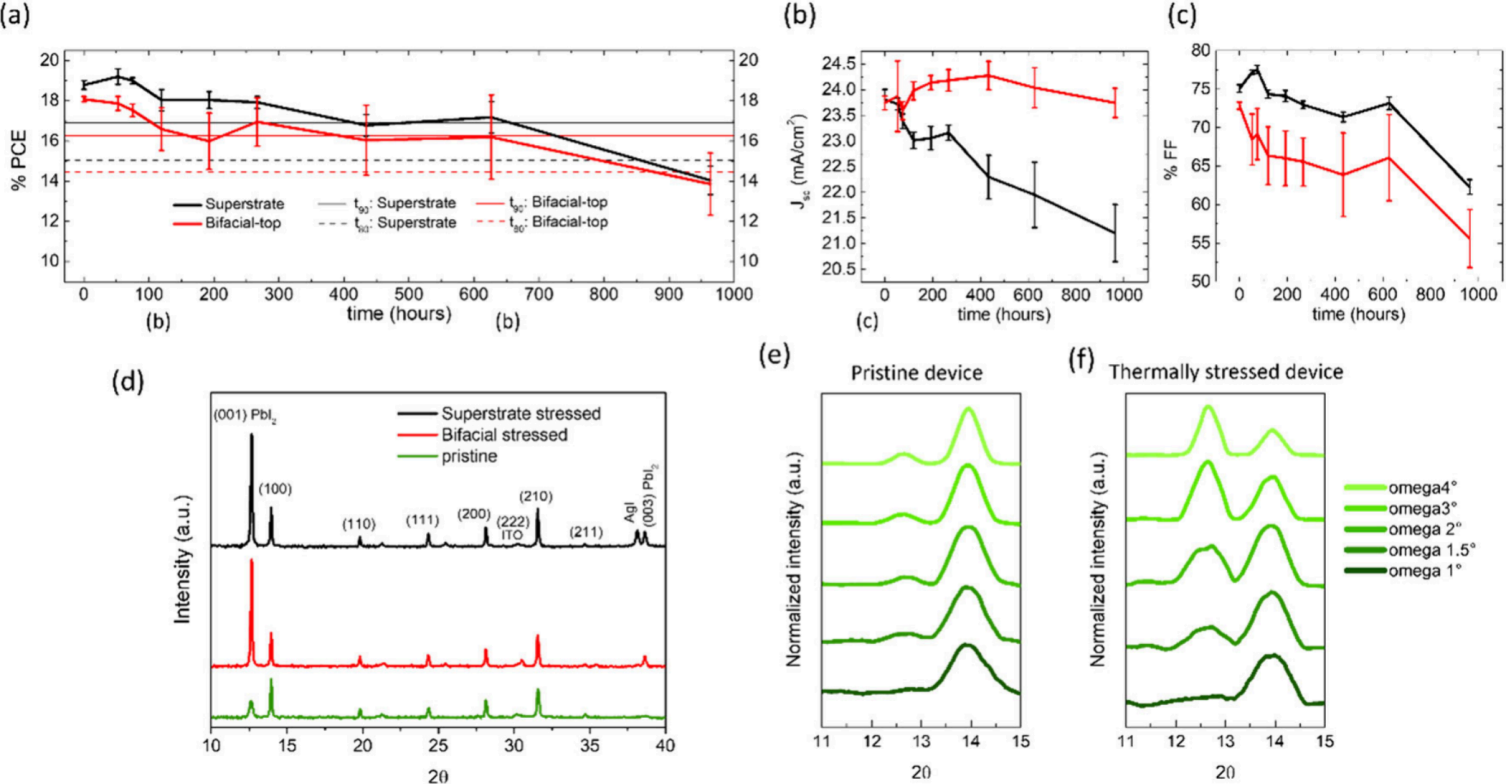
(a–c) Evolution of the PCE, *J*_sc_, and FF values of the representative Bi-PSC measured from top-illumination and corresponding superstrate PSC upon thermal stressing on a hot plate at 85 °C in a N_2_ atmosphere. The thin solid and dotted lines in panel (a) mark the 90% and 80% of initial PCE values, respectively of the two types of PSCs. The vertical error bars denote the standard deviation values. (d) X-ray diffraction pattern of the pristine ∼720 nm FAMAPI layer, as well as of the above thermally stressed bifacial and superstrate PSCs. (e–f) Grazing incidence X-ray diffraction (GIXRD) patterns of a pristine FAMAPI layer and thermally stressed superstrate device obtained at omega values ranging from 1° to 4°.

In conclusion, we demonstrated fully vacuum-deposited Bi-PSCs having a FAMAPI layer thickness up to ∼1.3 μm. With the optimal ITO and LiF layer thicknesses at the top-electrode, the Bi-PSCs exhibited higher *J*_sc_ under top-illumination than bottom illumination with a mean difference of more than 1 mA/cm^2^. The highest *J*_sc_ of 24.98 mA/cm^2^ was achieved under top-illumination in the Bi-PSC having the thickest, ∼1.3 μm, FAMAPI layer. The best Bi-PSC exhibited a PCE of 19.6% under top-illumination condition, and 18.71% under bottom illumination resulting in a bifaciality factor of approximately ∼0.95. Upon bifacial-illumination using simulated 1-Sun light as the main illumination and a white LED light as the albedo (rear) illumination, the above Bi-PSC demonstrated *J*_sc_ (output power density in mW/cm^2^) values of 25.78 (19.60), 27.92 (21.13), and 30.07 (22.63) mA/cm^2^ under albedo values of ∼0.06, ∼0.14, and ∼0.21, respectively. Even after employing a cover glass encapsulation at the top-electrode side, Bi-PSC still demonstrated higher *J*_sc_ from top-illumination than bottom-illumination. Both the superstrate and Bi-PSCs retained more than 80% of their initial PCE for more than 600 h when stressed on a hot plate at 85 °C inside a glovebox. This work demonstrates the efficacy of the thermal evaporation method in producing micrometer thick perovskite films for Bi-PSC application and of the top-electrode in producing high *J*_sc_ values in the Bi-PSCs under the primary AM 1.5 G monofacial illumination.

## Supporting Information Available

Experimental section; optical constants used in simulations; additional EQE spectra; *J–V* curves and contour plots of simulated *J*; cross-sectional SEM; Tauc plots; X-ray diffraction patterns; solar cell statistics; dark *J*–*V* curves; comparison of *J*_sc_ values of Bi-PSCs from the literature; and photography of a stressed device (PDF)
