# Supplementary material for: Vacuum-Deposited Bifacial Perovskite Solar Cells
Source: ACS Energy Lett. 2024 Aug 27;9(9):4587–95. doi: 10.1021/acsenergylett.4c01536 (PMC11406524; doi:10.1021/acsenergylett.4c01536)
Supplement: Supplementary file 1 — nz4c01536_si_001.pdf [file nz4c01536_si_001.pdf]

Vacuum-Deposited Bifacial Perovskite Solar Cells

Abhyuday Paliwal[1], Kassio P.S. Zanoni[1], Cristina Roldán-Carmona[1], Nathan Rodkey[1] and Henk J. Bolink[1,*]

* Corresponding authors: henk.bolink@uv.es
1. Instituto de Ciencia Molecular, Universidad de Valencia, Calle Catedrático José Beltrán 2, 46980 Paterna, Spain

SUPPORTING INFORMATION

Methods:

Chemicals:

CS-9, a high electron affinity p-dopant, Bathocuproine (BCP) and Methylammonium Iodide (MAI) were purchased from Lumtec. TaTm: ((N4,N4,N4,N4-tetra([1,1-biphenyl]-4-yl)-[1,1:4,1-terphenyl]-4,4-diamine) was purchased from Tokyo Chemical Industry (TCI). Formamidinium iodide (FAI) was bought from Greatcell. Fullerene (C_{60}) (purity > 99.95%) was purchased from Creaphys GmbH, and PbI_2 in form of beads (99.999% metal basis) was purchased from Alfa Aesar.

Fabrication of fully vacuum-deposited PSCs:

Bifacial PSC (Bi-PSC) device stack:

LiF 100 nm/ glass/ bottom-ITO 147 nm or 210 nm/ CS-9 2.5 nm/ TaTm 8.5 nm/ FAMAPI 720 nm, 880 nm or 1.3 µm/ C60 15 nm/ BCP 7.5 nm/ buffer ITO ~7 nm/ main harsh top-ITO ~140 nm/ Ag fingers 100 nm/ alumina 25 nm/ LiF 75 nm

Superstrate PSC device stack:

LiF 100 nm/ glass/ bottom-ITO 147 nm or 210 nm/ Cs-9 2.5 nm/ TaTm 8.5 nm/ FAMAPI 720 nm, 880 nm or 1.3 µm/ C60 15 nm/ BCP 7.5 nm/ Ag 100 nm/ alumina 25 nm

Note: For Bi-PSCs and superstrate PSCs having ~720 nm and 1.3 µm FAMAPI layers, the bottom-ITO thickness was ~147 nm, whereas, for Bi-PSCs and superstrate PSCs having ~880 nm FAMAPI layers, the bottom-ITO thickness was ~210 nm.

Fabrication of bifacial and superstrate PSCs:

Photolithographically patterned ITO coated glass substrates (irrespective of bottom-ITO thickness) were cleaned by following a standard procedure using soap, water, de-ionized water and isopropanol in a sonication bath, followed by UV treatment for 20 min. For device fabrication, the cleaned substrates samples were transferred to a nitrogen-filled glovebox (H_2O and O_2 < 0.1 ppm) containing the vacuum thermal evaporation chambers. All the films were thermally evaporated using appropriate shadow masks following the order of the stack of the photovoltaic devices in a pressure range of 10^{-6} mbar. The organic charge transport layers: CS-9, TaTm, C_{60} and BCP were evaporated in a dedicated thermal evaporator system at a rate of 0.2-0.3 Å/s. The co-evaporation process of the FAMAPI layer is described below. The top-ITO electrode in the case of Bi-PSCs was deposited by the pulsed laser deposition process and consisted of a thin, softly deposited ITO buffer layer followed by a thick, harshly deposited primary ITO layer. For depositing the Ag top electrode in superstrate devices and Ag fingers in Bi-PSCs, Ag was evaporated at a rate of 0.04 Å/s for the first 15 nm followed by the rate of ~ 0.1 Å/s for the remaining 75 nm. LiF antireflection layer was evaporated at 0.2 Å/s after encapsulation of the devices on both the sides of the device: on top of alumina and glass substrates in the case of Bi-PSCs, and on top of the glass substrate in the case of superstrate PSCs. A 25 nm alumina layer was deposited on all the PSCs using atomic layer deposition at a temperature of 40 °C. The active area of the devices defined as the overlapping area between the 'top Ag-electrode' or 'top-ITO + Ag fingers' and 'bottom-ITO' is 0.0825 cm^2.

FAMAPI co-evaporation:

FAMAPI film was grown by co-evaporating PbI_2 and MAI, and FAI. Rate of evaporation of PbI_2 and FAI were monitored using dedicated quartz crystal microbalance (QCMs) sensors close to the PbI_2 and FAI sources and manually held constant by adjusting their temperature throughout the evaporation process. Since control of MAI evaporation is not trivial (REF), the evaporation of MAI was controlled indirectly using a QCM sensor positioned close to the substrate holder, at a distance of less than 5 cm below it. This QCM sensor had the combined exposure of the fluxes of PbI_2, FAI and MAI, and the optimum value of the measured net rate of their evaporation was also manually held constant by adjusting the temperature of the MAI's source throughout the evaporation process.[1] On the other hand, the rates of PbI_2 and FAI were kept constant by their individual QCMs.[2] Thicknesses and thus the rates of all the organic and inorganic molecules were controlled by applying calibration factors to QCMs for each material. The latter was obtained

previously, by individually measuring the actual thickness of each layer deposited on glass using a mechanical profilometer (Ambios XP1) and then comparing it with the corresponding value given by the QCM.

Encapsulation of Bi-PSC with cover glass:

All the following steps were carried out inside a inside a N_2 glovebox. Bi-PSCs finished till alumina or LiF layers were coated (on the top-electrode side) with a UV-curable Eversolar AB302 paste from Everlight (thickness on micron scale). The clean cover glass slips were then carefully placed on top of the epoxy layer avoiding the formation of air-bubbles. The above samples were then placed under UV-light for ~5 minutes to cure the epoxy and cause firm adhesion of the cover glass onto the Bi-PSC.

Optical characterization and simulations:

The absorbance and transmittance spectrum were measured using a fiber optics based Avantes Avaspec2048 Spectrometer. A glass-substrate was used as a reference during the absorbance measurements of the FAMAPI layers. The reflectance measurements of the Bi-PSCs and superstrate PSCs were done in a system (QE-R) developed by by Enli Technology Co., Ltd.

Transfer matrix method-based absorptance and 1-R simulations were performed in a home-built code that uses the tmm package on a python-based IDE. The derivations of the formulas and calculations implemented to develop the tmm package can be found here.[3] The values of the optical constants (n,k) of the FAMAPI layer were approximately extrapolated from that of a slightly wider band gap perovskite composition in a reasonable way .

Structural characterization:

The XRD patterns of the FAMAPI layers or PSCs were measured in Bragg−Brentano geometry or grazing incidence geometry using appropriate optics on an Empyrean PANalytical powder diffractometer with a copper anode operated at 45 kV and 40 mA.

Electrical characterization:

The J-V curves for the solar cells were recorded using a Keithley 2612A Source Meter in a voltage range of -0.1 and 1.1 V, with 0.02 V steps and by integrating the signal for 20 ms after a 10 ms delay. The devices were illuminated under a Wavelabs Sinus 70 LED solar simulator. The

mismatch factors of the representative Bi-PSCs and the corresponding superstrate PSC are given in **Table S4**. The light intensity of the LED lamp was calibrated before the measurement using a calibrated Si reference diode (equipped with an infrared KG-05 cut off filter). The active area under illumination for both the superstrate and substrate devices was defined using a shadow mask and had a value of 0.05 cm^2. Bifacial illumination condition was simulated using fixed 1-Sun illumination from the Newport solar simulator along with variable intensity white light illumination from a commercially bought LED panel (**Figure S11**). Any constant intensity illumination from the LED was maintained via the application of a constant current to it via a Keithley sourcemeter. The J_{sc} of a Si reference diode (equipped with an infrared KG-05 cut off filter) was measured for the same varied illumination conditions of the white LED light (using a shadow mask) as were employed in the bifacial illumination of the Bi-PSCs (i.e., by applying the same set of current values to the LED). The above obtained J_{sc} values were compared to the EQE of the reference diode, to obtain the corresponding integrated current density values of the white light at different illumination conditions as represented by the following equation:

$\int EQE_{Si}(\lambda) \times J(\lambda)\ (mAcm^{-2}nm^{-1}) \times d\lambda = J_{sc}$ measured at different illumination conditions

Where $\int J(J) \times d\lambda$ is the integrated current density values of the white light at different illumination conditions. To obtain the albedo values, we divided the above obtained integrated J values (in mA/cm^2) with the current density value corresponding to the detailed balance limit of a solar cell (a step-function absorption profile) having an absorber with a band gap of 1.53 eV.

The EQE measurements a system (QE-R) developed by Enli Technology Co., Ltd. The system was calibrated, and the solar spectrum mismatch was corrected using a calibrated Silicon reference cell. All the PSCs fabricated in this work showed a mismatch in the J_{sc} defined as the difference between the average J_{sc} value of a PSC obtained from the J-V curves under simulated 1-Sun measurement (obtained from multiple devices) and the integrated J_{sc} value obtained from their EQE spectrum. The mismatch in the J_{sc} values ranged from 0.9 -1.2 mA/cm^2 (**Table S4, Table S5, Table S6**).

Supplementary figures and tables:

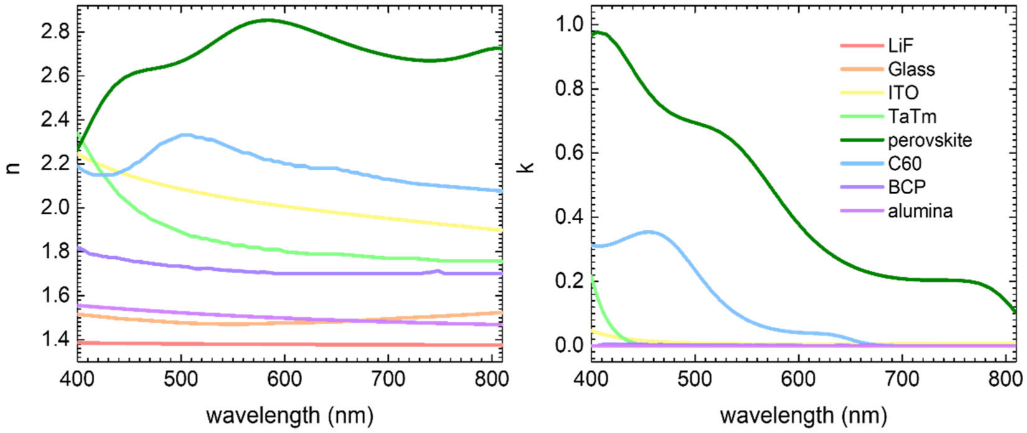

Figure S1. Optical constants (n, k) used for transfer matrix-based optical simulations.

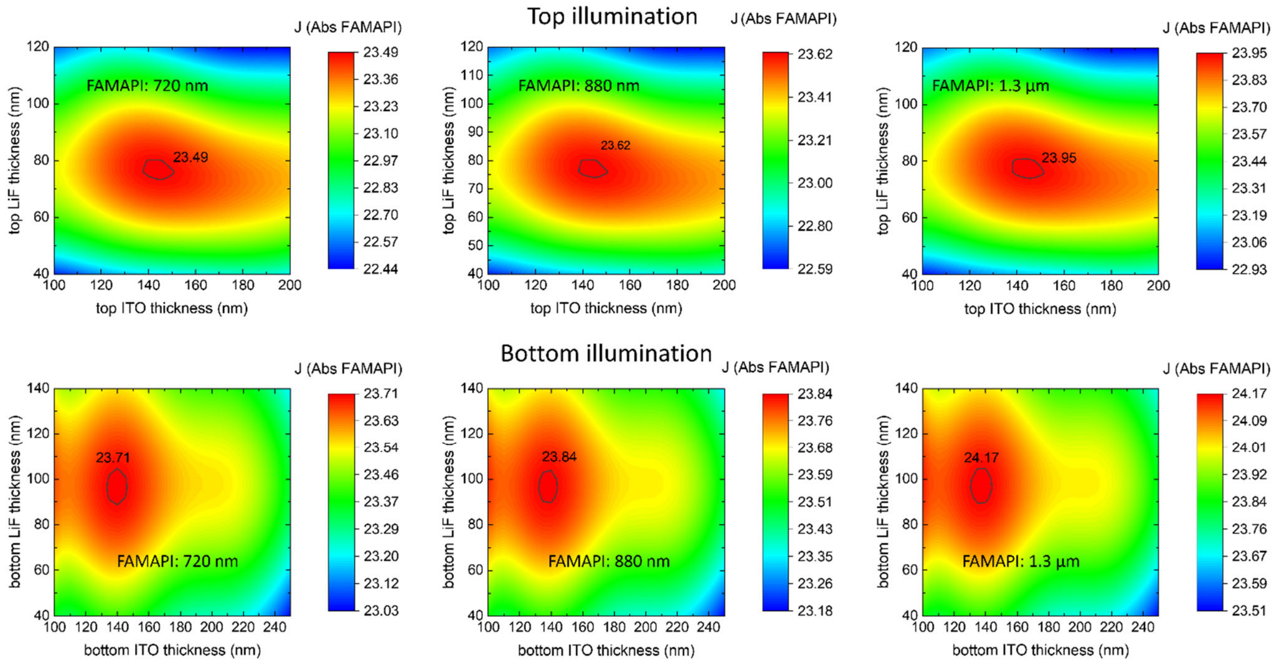

Figure S2. Contour plots of simulated J (Abs FAMAPI) values of the Bi-PSCs having different thicknesses of the FAMAPI layer (720 nm, 880 nm and 1.3 μm), for a range of thicknesses of LiF and ITO layers (thickness: ITO > 100 nm) at both top and bottom-electrodes under top and bottom-illumination conditions, respectively. The solid line in every contour plot marks the region exhibiting the highest J (Abs FAMAPI) value. It can be observed that the optimal values of LiF and ITO layers of both the top and bottom-electrodes are not influenced by perovskite thickness in the Bi-PSC. Note that optimal thickness values of bottom ITO and bottom LiF layers were used for simulating the J (Abs FAMAPI) from top-illumination, and optimal thickness values of top ITO and top LiF layers were used for simulating the J (Abs FAMAPI) from bottom-illumination.

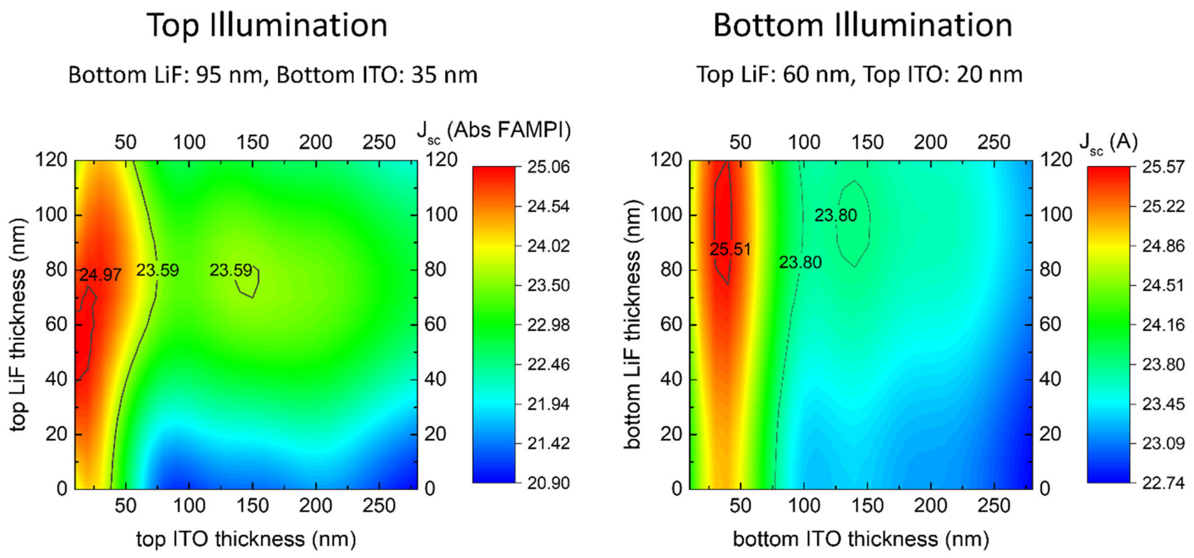

Figure S3. Contour plots of simulated J (Abs FAMAPI) values of the Bi-PSCs for a wider range of thicknesses of LiF and ITO layers at both top and bottom-electrodes under top and bottom-illumination conditions, respectively. Note that the optimum thicknesses of LiF and ITO layers at the top-electrode are 60 nm and 20 nm, respectively, and at the bottom-electrode are 95 nm and 35 nm, respectively. In the above contour plots of simulated J (Abs FAMAPI) values, we have not only marked the region exhibiting the absolute highest J (Abs FAMAPI) value, but also the regions of the highest J (Abs FAMAPI) value such that the magnitude of at least a part of the corresponding ITO thicknesses is more than 100 nm. Note that optimal thickness values of bottom ITO and bottom LiF layers were used for simulating the J (Abs FAMAPI) from top-illumination,

and optimal thickness values of top ITO and top LiF layers were used for simulating the J (Abs FAMAPI) from bottom-illumination.

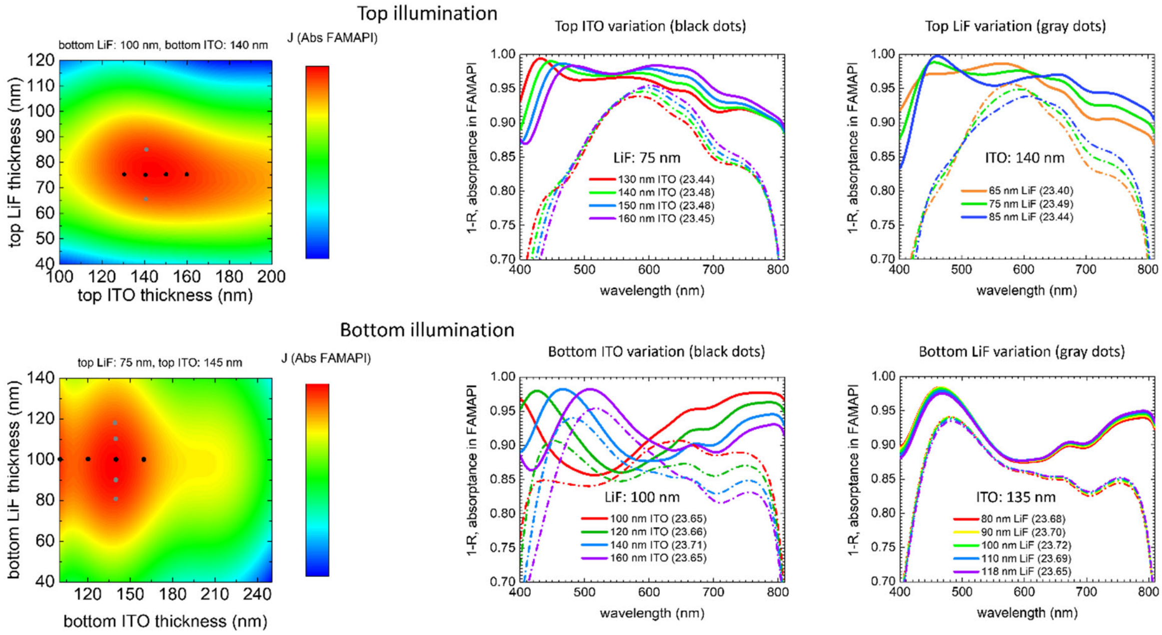

Figure S4. Contour plots of simulated J (Abs FAMAPI) values of the Bi-PSCs for a wide range of thicknesses of LiF and ITO layers at both top and bottom-electrodes under top and bottom-illumination conditions, respectively (these contour plots are the same as in **Figure 1b,c** of the main text). Simulated 1-R spectra of the Bi-PSC as well as the absorptance of the FAMAPI layer when the Bi-PSC has optimal and sub-optimal thicknesses of the LiF and ITO layers at both the top and bottom-electrodes under top and bottom-illumination conditions, respectively. At both the top and bottom electrodes (under top and bottom-illumination conditions, respectively), the thickness of either the ITO layer (black dots in contour plots) or the LiF layer (gray dots) are varied, and the resultant simulated 1-R spectra of the Bi-PSC as well as the absorptance of the FAMAPI layer are presented in the above figure. Note that simulated J (Abs FAMAPI) value of every case is given in the legend (inside round brackets) of its panel alongside its name. The influence of the shape of the AM 1.5 G photon flux spectrum on current generation can be visualized by comparing the spectral evolution of the simulated absorptance spectrum of the FAMAPI layer (due to variation in any layer thickness) with the evolution of the corresponding simulated current density values; in particular from the fact that while the evolution of the spectra

follows a specific trend, it doesn't lead to a strict increment or decrement in the J (Abs FAMAPI) values.

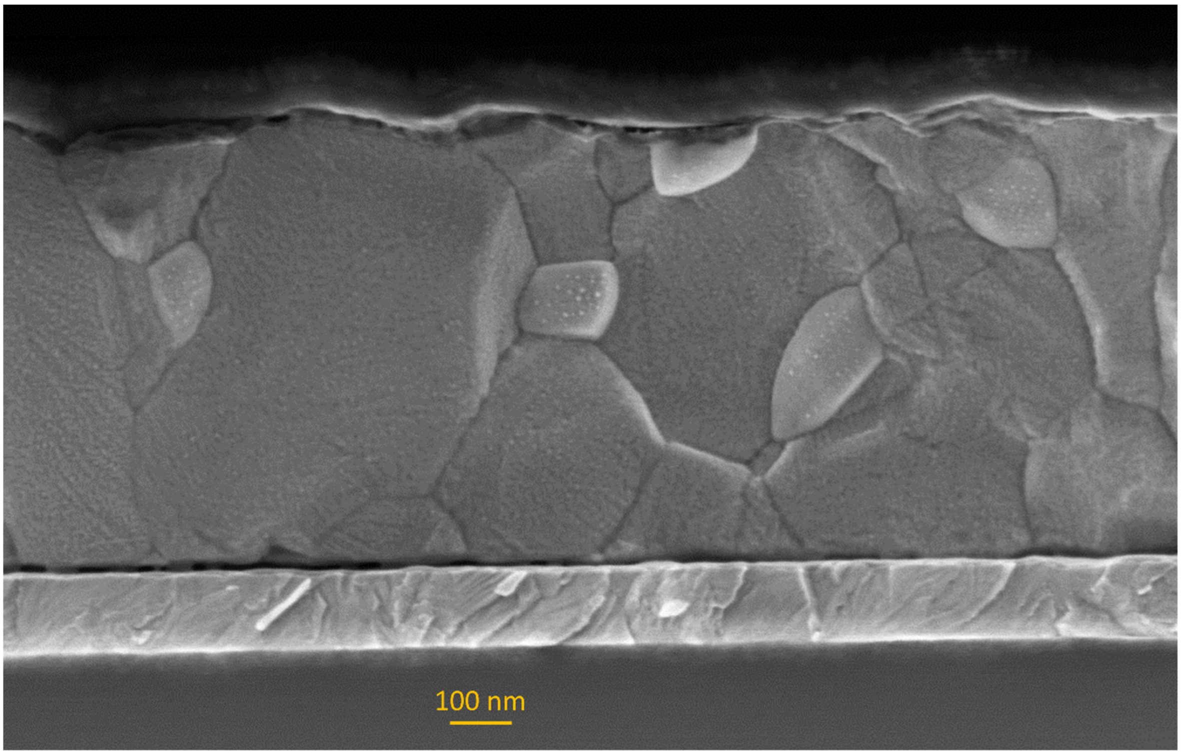

Figure S5. Large cross-sectional SEM image of the Bi-PSC having ~720 nm FAMAPI layer.

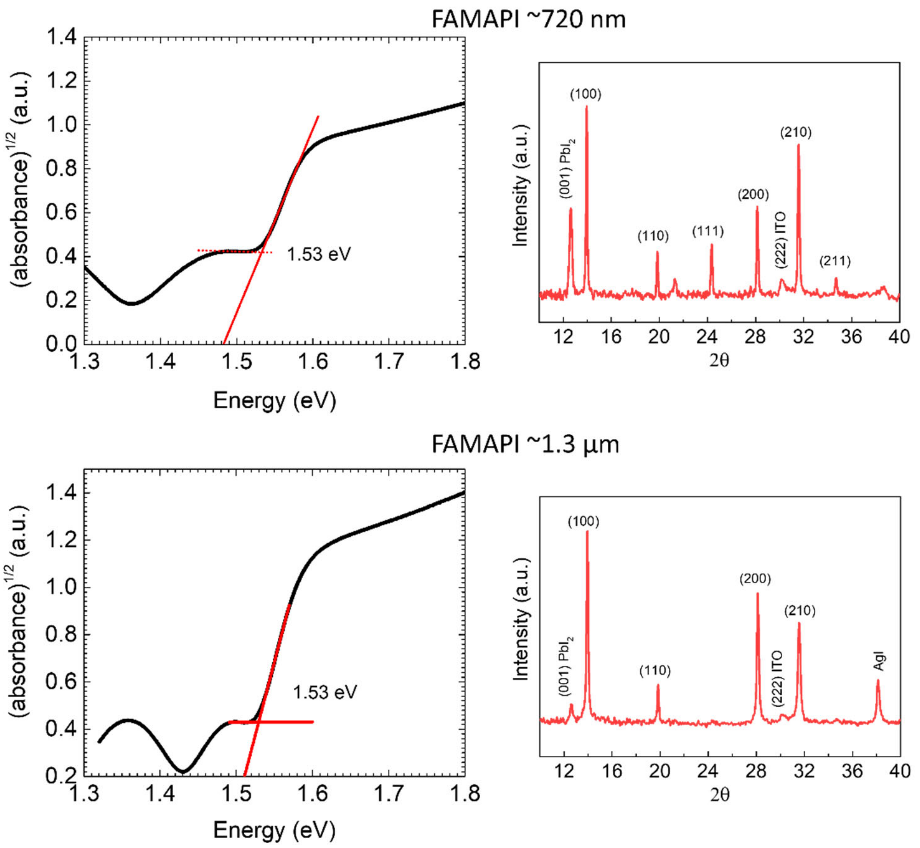

Figure S6. Tauc plots and X-ray diffraction patterns of the FAMAPI layers having thicknesses of ~720 nm and ~1.3 µm. The two FAMAPI layers exhibit the same band gap of ~1.53 eV as revealed by their respective Tauc plots. The method employed for deriving the band gap from the Tauc plot is taken from the publication of Makula et. al. [4] On the other hand, a preliminary observation of the X-ray diffraction patterns reveal that there's a difference in the 'texture' of the two FAMAPI layers as indicated by their different intensity ratios of (200) and (210) peaks, i.e., $I_{(200)}/I_{(210)}$ and (100) and (111) peaks, i.e., $I_{(100)}/I_{(111)}$.[2,5] Note that the AgI peak is seen in the diffraction pattern of ~1.3 µm FAMAPI layer as its diffractogram was obtained from the measurement of the corresponding superstrate sample.

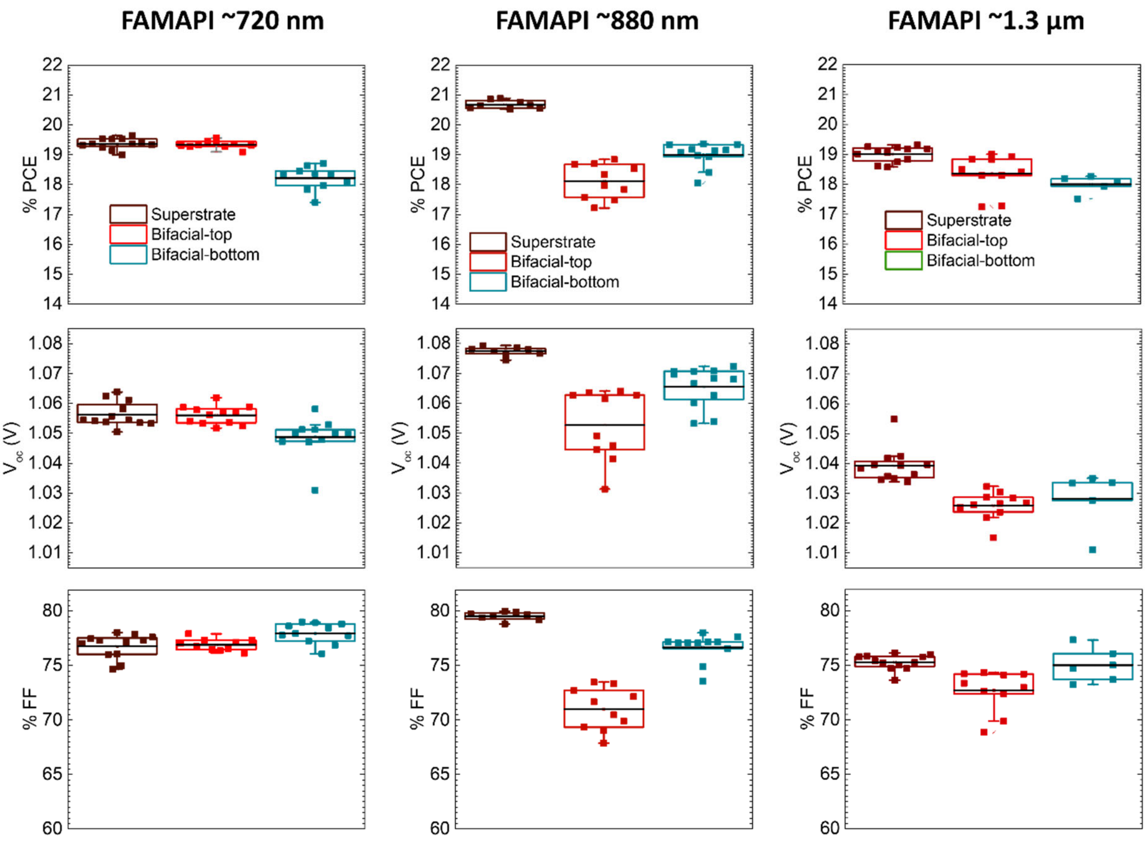

Figure S7. PCE parameters (except J_{sc}) of the bifacial and superstrate PSCs having different thicknesses of FAMAPI layers: ~720 nm, ~880 nm, and 1.3 µm, under simulated 1-Sun illumination obtained from reverse scan (V_{oc} to J_{sc}).

Table S1. PCE parameters obtained under forward (fwd.) and reverse (rev.) scans of champion bifacial and superstrate PSCs having different FAMAPI layer thicknesses.

FAMAPI thickness (nm)	Device	% PCE fwd. (rev.)	V_{oc} (V) fwd. (rev.)	J_{sc} (mA/cm^2) fwd. (rev.)	% FF fwd. (rev.)
**~720**	Superstrate	19.0 (19.5)	1.060 (1.064)	24.08 (24.13)	74.6 (76.1)
**~720**	Bifacial-top	18.3 (19.6)	1.040 (1.053)	23.89 (23.82)	73.5 (77.9)
**~720**	Bifacial-bottom	17.4 (18.7)	1.039 (1.051)	22.49 (22.55)	74.6 (78.9)
**~880**	Superstrate	19.6 (20.6)	1.063 (1.072)	24.25 (24.14)	76.2 (79.5)
**~880**	Bifacial-top	18.4 (18.6)	1.047 (1.052)	24.79 (24.83)	70.8 (71.1)
**~880**	Bifacial-bottom	18.0 (19.4)	1.060 (1.071)	23.41 (23.18)	72.5 (78.0)
**~1300**	Superstrate	18.0 (19.3)	1.030 (1.040)	24.06 (24.50)	72.4 (75.8)
**~1300**	Bifacial-top	17.6 (19.0)	1.016 (1.025)	24.81 (24.98)	69.6 (74.2)
**~1300**	Bifacial-bottom	17.2 (18.3)	1.022 (1.028)	22.95 (23.35)	73.3 (76.1)

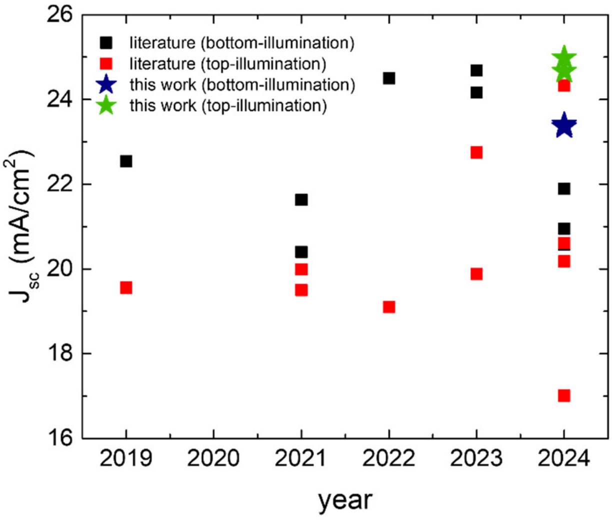

Figure S8. Comparison of J_{sc} values of Bi-PSCs of this work and from the literature under monofacial, simulated 1-Sun top and bottom-illumination of the Bi-PSCs.[6–13]

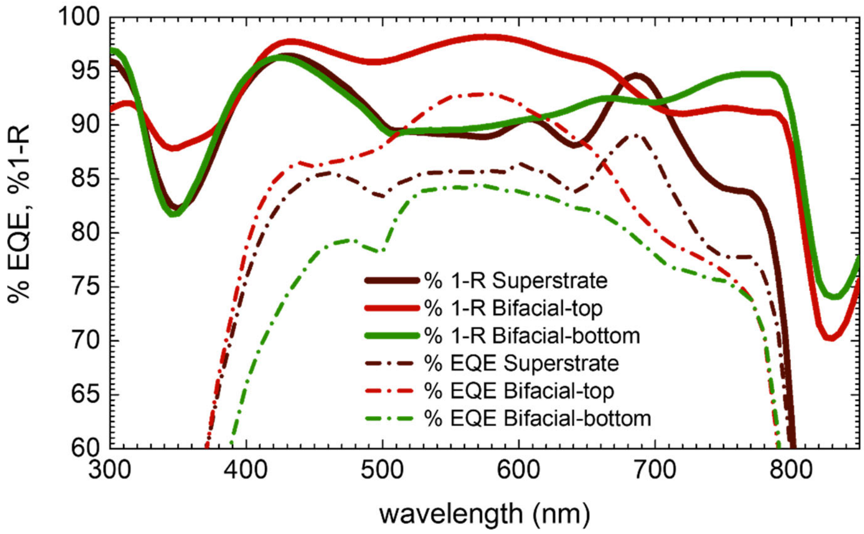

Figure S9. Comparison of measured 1-reflectance (1-R) and EQE spectra of the representative Bi-PSCs (having ~720 nm FAMAPI layer) under top and bottom-illumination conditions as well as the corresponding superstrate device.

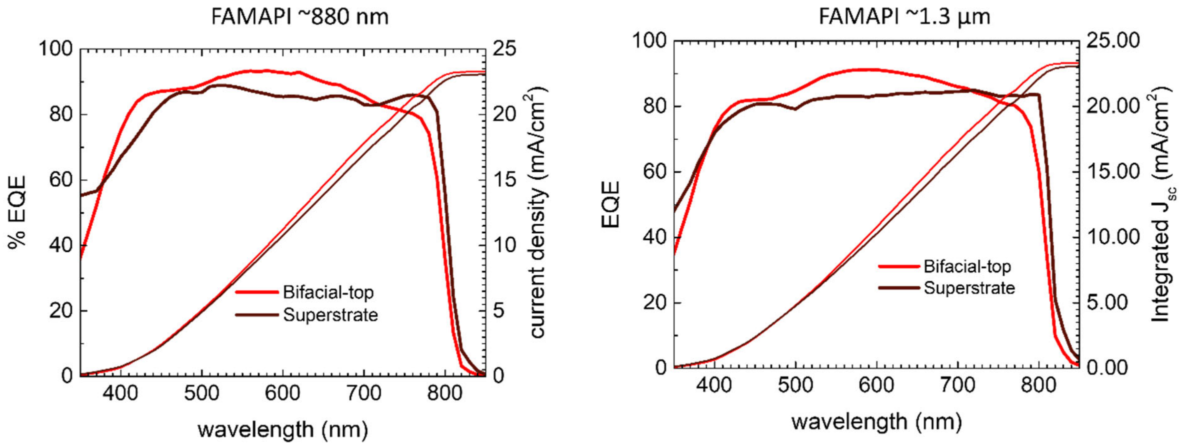

Figure S10. EQE spectra of the bifacial and superstrate PSCs having ~880 nm and ~1.3 µm FAMAPI layers. Note that the thicknesses of bottom-ITO layers in these devices are 210 nm, and 147 nm, respectively.

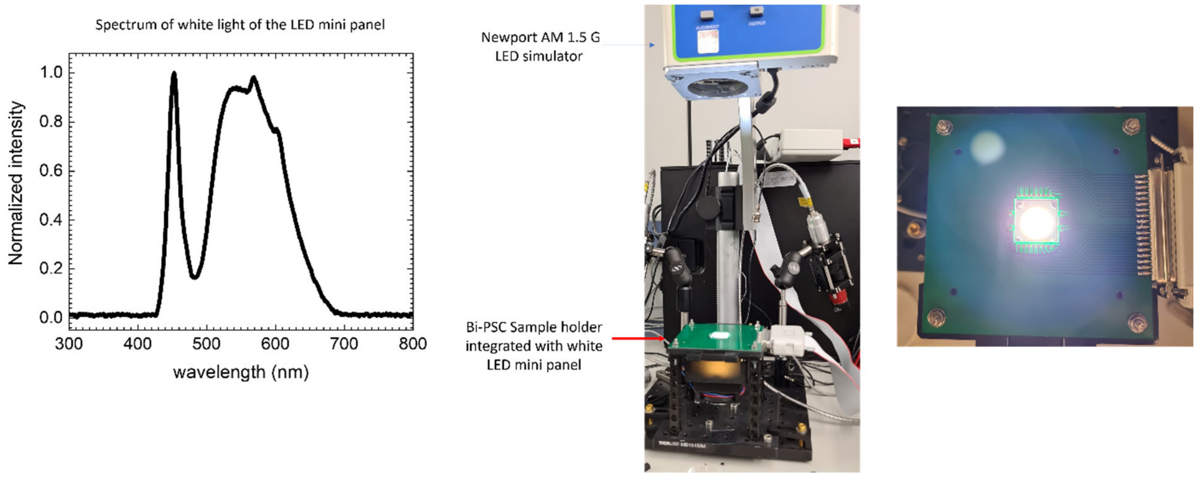

Figure S11. On the left, the spectrum of the white light of the LED mini panel which was used for simulating albedo in the bifacial-illumination of the Bi-PSCs. In the center, the photograph of the bifacial-illumination setup. On the right, the photograph of the white light LED mini panel equipped Bi-PSC sample holder.

Table S2. Output power density values of the representative Bi-PSC (having ~720 nm FAMAPI layer) as extracted from the J-V curves under forward (fwd.) and reverse (rev.) scans under various bifacial-illumination conditions (stimulated 1-Sun and albedo from the white-light illumination), along with the corresponding device performance parameters.

albedo	Output power density (mW/cm^2) fwd. (rev.)	V_{oc} (V) fwd. (rev.)	J_{sc} (mA/cm^2) fwd. (rev.)	% FF fwd. (rev.)
**0.000**	18.19 (18.40)	1.036 (1.035)	23.99 (24.00)	73.2 (74.0)
**0.027**	18.68 (19.05)	1.037 (1.036)	24.78 (24.77)	72.6 (74.2)
**0.063**	19.42 (19.90)	1.038 (1.037)	25.80 (25.78)	72.4 (74.4)
**0.138**	20.98 (21.56)	1.041 (1.040)	27.94 (27.92)	72.2 (74.3)
**0.213**	22.57 (23.05)	1.041 (1.041)	30.12 (30.07)	72.0 (73.6)

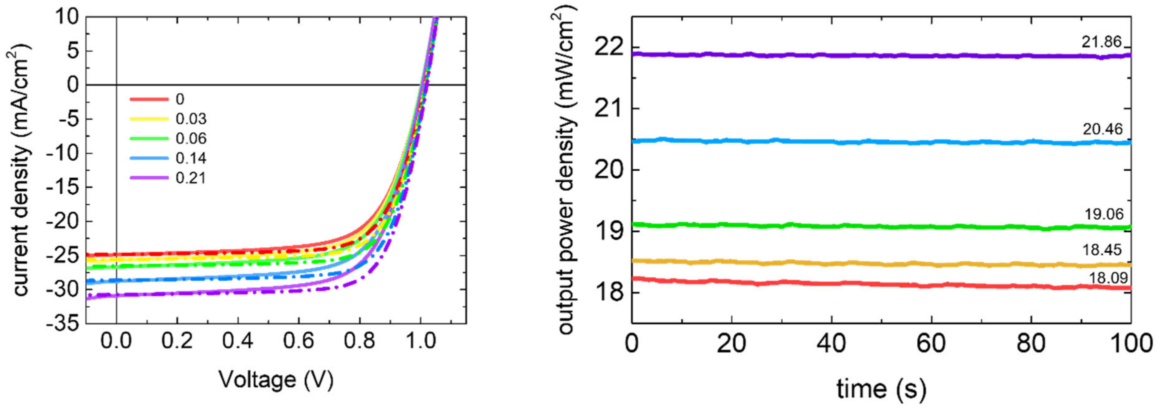

Figure S12. On the left, the J-V curves obtained from forward scan (solid) and reverse scan (dotted) of the Bi-PSC having ~1.3 µm FAMAPI layer under bifacial-illumination conditions comprising: a constant simulated 1-Sun illumination and albedo from the white-light illumination ranging from 0 to ~0.21 (rounded off to second place after decimal). On the right, the maximum power point tracking of the representative Bi-PSC under bifacial-illumination conditions as described in the left-panel. The numbers in the panel indicate the power density generated by the Bi-PSC under different bifacial-illumination conditions at the end of 100 seconds.

Table S3. Output power density values of the Bi-PSC having ~1.3 μm FAMAPI layer as extracted from the J-V curves under forward (fwd.) and reverse (rev.) scans under various bifacial-illumination conditions (stimulated 1-Sun and albedo from the white light illumination), along with the corresponding device performance parameters.

albedo	Output power density (mW/cm^2) fwd. (rev.)	V_{oc} (V) fwd. (rev.)	J_{sc} (mA/cm^2) fwd. (rev.)	% FF fwd. (rev.)
**0.000**	16.93 (18.06)	1.004 (1.014)	24.86 (24.83)	67.8 (71.7)
**0.027**	17.47 (18.86)	1.003 (1.015)	25.66 (25.56)	67.9 (72.7)
**0.063**	18.16 (19.68)	1.003 (1.016)	26.66 (26.54)	67.9 72.9)
**0.138**	19.60 (21.28)	1.005 (1.018)	28.75 (28.62)	67.8 (73.0)
**0.213**	21.03 (22.89)	1.007 (1.020)	30.87 (30.73)	67.6 (73.0)

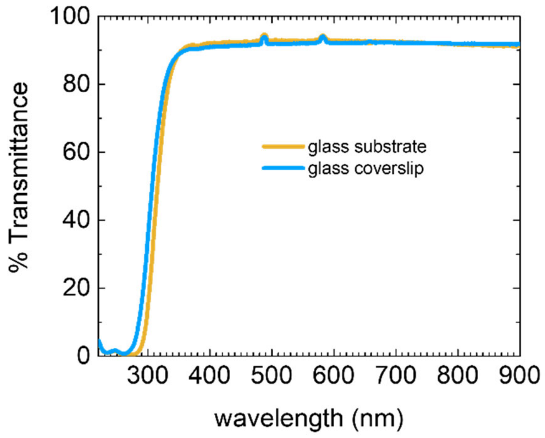

Figure S13. Transmittance spectrum of a general glass-substrate and cover glass used for encapsulating the Bi-PSC.

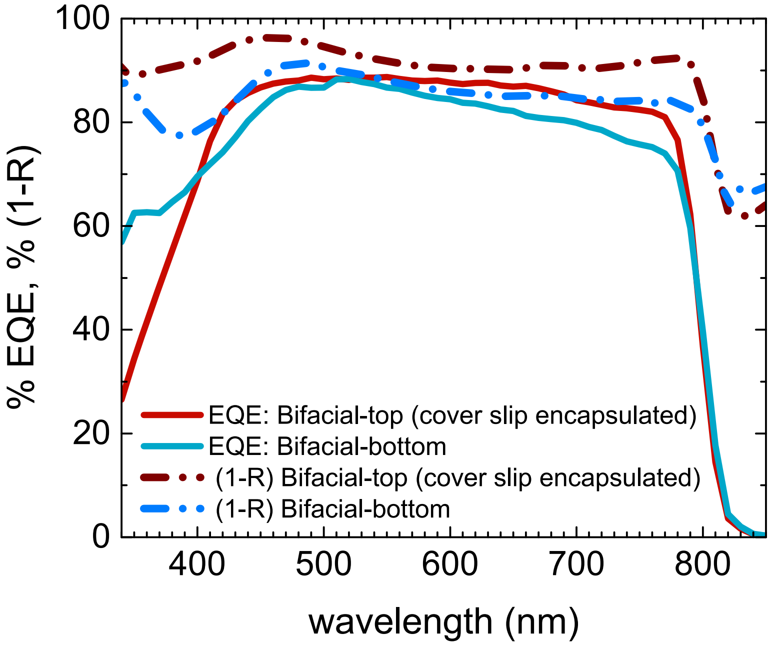

Figure S14. Comparison of EQE spectra, and 1-R spectra of the cover glass encapsulated Bi-PSC under top and bottom-illumination conditions (or from the top and bottom-electrodes).

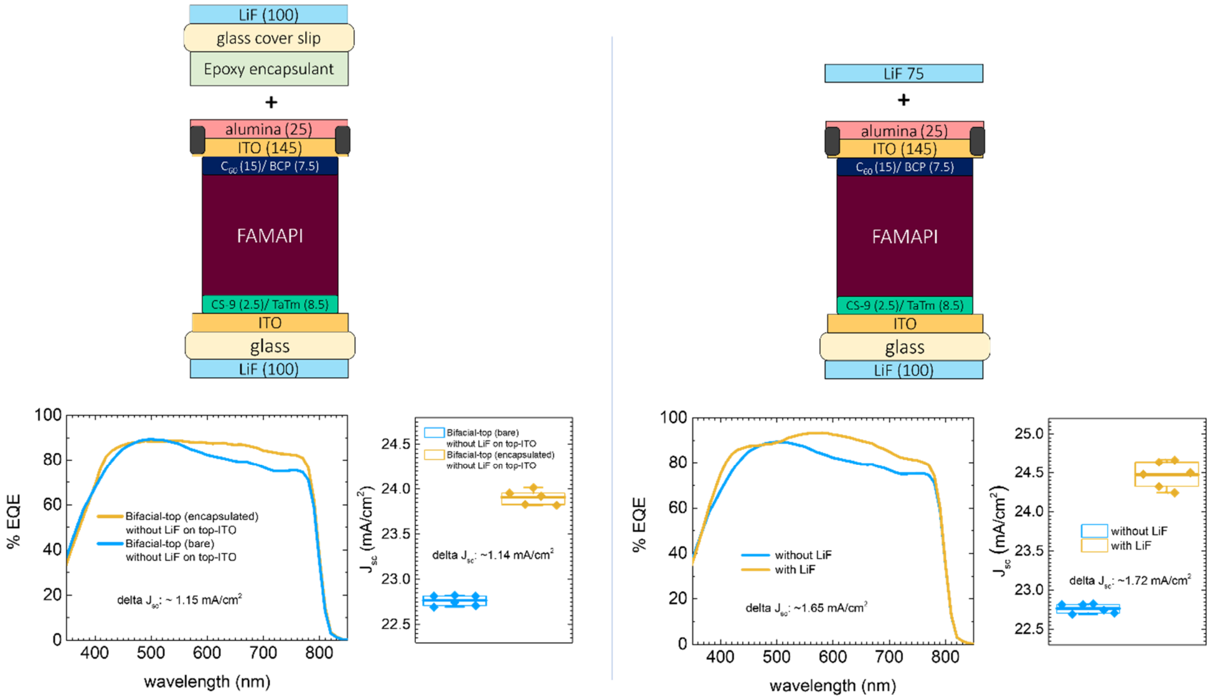

Figure S15. Comparison of improvement in the EQE and J_{sc} values (under top-illumination) of the Bi-PSC finished till the alumina layer when cover glass encapsulation along with a 100 nm LiF anti-reflection layer is added over (left side), and a single, optimal ~75 nm LiF layer is added over (right side)

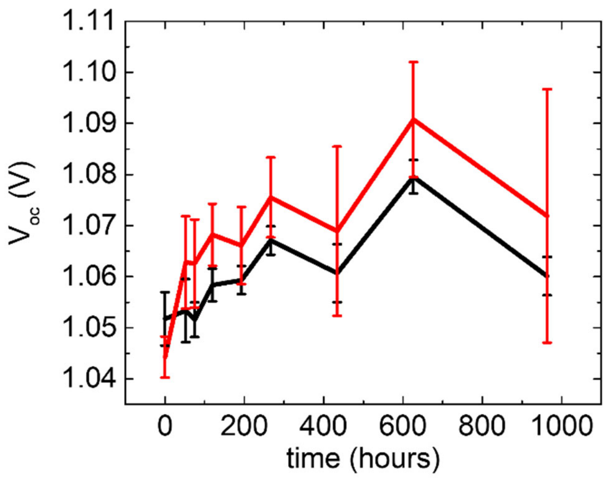

Figure S16. Evolution of the V_{oc} values of the representative Bi-PSC measured from top-illumination and corresponding superstrate PSC upon thermal stressing on a hot plate at 85 °C in N_2 atmosphere. The vertical error bars denote the standard deviation values.

Table S4. Average J_{sc} values of the representative Bi-PSC obtained under top and bottom-illumination conditions and the corresponding superstrate PSC from their respective J-V curves under simulated 1-Sun illumination, the corresponding integrated J_{sc} values derived from their respective EQE spectrum, and the calculated mismatch factor values.

device	Avg J_{sc} (mA/cm^2) J-V: 1-Sun illumination	Integrated J_{sc} from EQE (mA/cm^2)	Delta Jsc (mA/cm^2)	Mismatch factor
**Superstrate**	23.89	22.98	0.91	0.1%
**Bifacial-top**	23.8	22.89	0.91	0%
**Bifacial-bottom**	22.26	21.33	0.93	1%

Table S5. Average J_{sc} values of the Bi-PSC having ~880 nm FAMAPI layer obtained under top and bottom-illumination conditions (with and without cover-glass encapsulation) and the corresponding superstrate PSC from their respective J-V curves under simulated 1-Sun illumination, and the corresponding integrated J_{sc} values derived from their respective EQE spectrum.

device	Avg J_{sc} (mA/cm^2) J-V: 1-Sun illumination	Integrated J_{sc} from EQE (mA/cm^2)	Delta Jsc (mA/cm^2)
**Superstrate**	24.23	23.07	1.16
**Bifacial-top**	24.47	23.28	1.19
**Bifacial-bottom**	23.25	22.02	1.23
**Bifacial-top (encapsulated)**	23.91	22.72	1.19

Table S6. Average J_{sc} values of the Bi-PSC having ~1.3 µm FAMAPI layer obtained under top and bottom-illumination conditions and the corresponding superstrate PSC from their respective J-V curves under simulated 1-Sun illumination, and the corresponding integrated J_{sc} values derived from their respective EQE spectrum.

device	Avg J_{sc} (mA/cm^2) J-V: 1-Sun illumination	Integrated J_{sc} from EQE (mA/cm^2)	Delta Jsc (mA/cm^2)
**Superstrate**	24.28	23.09	1.19
**Bifacial-top**	24.59	23.33	1.26

References:

[1] K. P. S. Zanoni, L. Martínez-Goyeneche, C. Dreessen, M. Sessolo, H. J. Bolink, *Solar RRL* **2023**, *7*, 2201073.

[2] A. Paliwal, K. P. S. Zanoni, C. Roldán-Carmona, M. A. Hernández-Fenollosa, H. J. Bolink, *Matter* **2023**, *6*, 3499.

[3] S. J. Byrnes, *arXiv preprint arXiv:1603.02720* **2016**.

[4] P. Makuła, M. Pacia, W. Macyk, *How to correctly determine the band gap energy of modified semiconductor photocatalysts based on UV–Vis spectra*, Vol. 9, ACS Publications, **2018**, pp. 6814–6817.

[5] L. Gil-Escrig, C. Dreessen, I. C. Kaya, B. S. Kim, F. Palazon, M. Sessolo, H. J. Bolink, *ACS Energy Lett* **2020**, *5*, 3053.

[6] Z. Song, C. Chen, C. Li, S. Rijal, L. Chen, Y. Li, Y. Yan, *Sustain Energy Fuels* **2021**, *5*, 2865.

[7] J. Heo, I. Jung, H. Park, J. H. Han, H. Kim, H. Park, J. Park, H. Jeon, K. Lee, H. J. Park, *Adv Opt Mater* **2022**, *10*, 2101696.

[8] C. Zhang, M. Chen, F. Fu, H. Zhu, T. Feurer, W. Tian, C. Zhu, K. Zhou, S. Jin, S. M. Zakeeruddin, *Energy Environ Sci* **2022**, *15*, 1536.

[9] D. Chen, S. Pang, L. Zhou, X. Li, A. Su, W. Zhu, J. Chang, J. Zhang, C. Zhang, Y. Hao, *J Mater Chem A Mater* **2019**, *7*, 15156.

[10] J. Zhang, X.-G. Hu, K. Ji, S. Zhao, D. Liu, B. Li, P.-X. Hou, C. Liu, L. Liu, S. D. Stranks, *Nat Commun* **2024**, *15*, 2245.

[11] N. Rodkey, K. P. S. Zanoni, M. Piot, C. Dreessen, R. Grote, P. Carroy, J. E. Sebastian Alonso, A. Paliwal, D. Muñoz, H. J. Bolink, *Adv Energy Mater* **2024**, 2400058.

[12] H. Gu, C. Fei, G. Yang, B. Chen, M. A. Uddin, H. Zhang, Z. Ni, H. Jiao, W. Xu, Z. Yan, J. Huang, *Nat Energy* **2023**, *8*, 675.

[13] Q. Jiang, Z. Song, R. C. Bramante, P. F. Ndione, R. Tirawat, J. J. Berry, Y. Yan, K. Zhu, *Joule* **2023**, *7*, 1543.
